# An exploratory qualitative assessment of factors influencing childhood vaccine providers' intention to recommend immunization in the Netherlands

**DOI:** 10.1186/1471-2458-12-128

**Published:** 2012-02-14

**Authors:** Liesbeth Mollema, Jojet M Staal, Jim E van Steenbergen, Theo GWM Paulussen, Hester E de Melker

**Affiliations:** 1 Centre for Infectious Disease Control, National Institute for Public Health and the Environment (RIVM), Bilthoven, the Netherlands; 2 Faculty of Earth and Life Sciences, VU University Amsterdam, Amsterdam, the Netherlands; 3 Netherlands Organization for Applied Scientific Research (TNO), Leiden, the Netherlands

## Abstract

**Background:**

Under the Dutch national immunization program (NIP), childhood vaccination is not mandatory, but its recommendation by childhood vaccine providers (CVP) is important for maintaining high vaccination coverage. We therefore examined factors related to providers' intentions to recommend vaccinations to parents of young children.

**Methods:**

We conducted four focus group discussions with nurses and physicians who provide vaccines to children 0-4 years old in diverse regions of the Netherlands. Three groups represented CVPs at child welfare centers (CWCs) serving the general population, with the fourth representing anthroposophical CWCs. Elements of the Theory of Planned Behaviour (TPB) were used to design the groups; thematic analysis was used to structure and analyze the dataset.

**Results:**

Four main themes emerged, including 1) perceived responsibility: to promote vaccines and discuss pros and cons with parents (although this was usually not done if parents readily accepted the vaccination); 2) attitudes toward the NIP: mainly positive, but doubts as to NIP plans to vaccinate against diseases with a low perceived burden; 3) organizational factors: limited time and information can hamper discussions with parents; 4) relationship with parents: crucial and based mainly on communication to establish trust. Compared to CVPs at standard CWCs, the anthroposophical CWCs spent more time communicating and were more willing to adapt the NIP to individual cases.

**Conclusions:**

Our qualitative assessment provides an overview of beliefs associated with providers' intention to recommend vaccinations. They were motivated to support the NIP, but their intentions to recommend vaccinations were affected by the perceived relevance of the vaccines, practical issues like limited time and by certain types of resistant parents. These results will inform future studies to test the magnitude and relative impact of these factors.

## Background

Childhood vaccine providers (CVPs) are key figures in implementation of an effective vaccination program [1-3]. Perceived by parents as the most important source for information about vaccines [4,5], they are well-positioned to address parental uncertainties and misconceptions. In the Netherlands, the National Institute for Public Health and the Environment (RIVM) manages the national immunization program (NIP) and supplies related information to CVPs and parents. Its recent introduction of vaccination against human papillomavirus (HPV) in the NIP and vaccination against Influenza A (H1N1) during the pandemic have stirred discussion and dissension [6]. As CVPs communicate directly with parents, it is crucial to maintain their support of the current and future NIP. Started in 1957, the program is non-mandatory, free of charge and currently includes vaccines against twelve target diseases. In recent years, vaccination coverage for infants ranged from 94.5% for diphtheria, pertussis, tetanus and polio (DTaP-IPV) to 96.0% for measles, mumps and rubella (MMR) (cohort 2005; reporting year 2008) [7]. Children of 0-4 years old receive vaccinations and other care at widespread child welfare centers (CWCs), while older children are served by municipal public health services. CWC care for each child begins with a home visit by a nurse and proceeds with vaccination and consults alternated by physicians and nurses on a scheduled basis. Instead of the standard CWC, parents may opt for a CWC based on anthroposophy, a spiritual philosophy founded by Rudolf Steiner [8].

On October 2011, universal hepatitis B vaccination was added to the NIP. Other new components are foreseen, such as varicella and rotavirus vaccine [9]. Given the central position of the CVPs, more insight is needed regarding the social and psychological determinants of their intentions to recommend current and future vaccinations to parents. Their support is important for Dutch policy makers who face the challenge of maintaining high coverage rates. Studies elsewhere have explored factors associated with CVP attitudes [10-12], but differences in health care systems, vaccination programs and cultures must be taken into account [13]. In the Netherlands, just one study has addressed the issue [14]. It was a quantitative study and our effort was to elaborate on its findings by means of focus group discussions with CVPs working with children of 0-4 years old. Our objective was to examine the factors behind their intentions to recommend current and future vaccinations to parents.

## Methods

### Study participants

Three focus groups were conducted with CVPs from standard CWCs, plus a fourth with anthroposophical CVPs. Two of the standard groups included 8 and 6 physicians, respectively and a third included 3 physicians and 3 nurses. The anthroposophical group included 5 physicians, making a total of 25 study participants. Focus groups were chosen as the approach because interaction in a group setting was likely to produce more data and insights than individual interviews. Listening to others' verbalized experiences of vaccination practices stimulates memories, ideas and experiences among participants.

For the fourth focus group, an anthroposophical physician recruited like-minded colleagues. The participants were recruited from various regions to represent the diversity of CWC populations (i.e., urban/rural, high/low education, non-Dutch/Dutch, people with varied motives for vaccine refusal). Part of the mideastern province of Gelderland was selected based on its relatively high number of inhabitants who refuse vaccination on religious grounds [7]. In the midnorthern and midwestern provinces of North-Holland and South-Holland, we selected urban and sub-urban regions in which CVPs encounter more migrants and also more vaccine refusal on non-religious grounds, e.g., support of an organization critical of vaccination or support of alternative medicine such as anthroposophy or homeopathy. We contacted staff of two CWCs in Gelderland and ten in South-Holland, providing an invitation letter for their employees. In Gelderland, additional invitation letters were sent via the regional mailing list of the Dutch Association for Physicians. Any CVP interested in participation could apply by e-mail. To represent North-Holland, a physician involved in vaccination-related expert committees recruited a group of colleagues from several CWCs.

All participants administered NIP vaccines to children 0-4 years of age at their own CWC. Participants were offered travel allowance and a gift voucher of €10,- as a token of gratitude. Only the moderator and assistant had access to the gathered data. Anonymity of statements in the final report was guaranteed. Informed consent or approval by an ethics committee was not required.

### Theoretical framework

Focus group design was based on elements of the Theory of Planned Behaviour (TPB) [15], which has been widely used to understand physicians' immunization behaviour [16-19]. The theory assumes that *intention *is a direct antecedent of *behaviour*. Three constructs lead to the formation of *a behavioural intention*, including 1) *Attitude: *behavioural beliefs produce a favourable or unfavourable attitude toward the behaviour; 2) *Social norms*: normative beliefs result in perceived social pressure; 3) *Perceived behavioural control*: the perceived ease or difficulty of performing the behaviour [20].

### Procedure

Our focus groups were semi-structured, with minimal steering to allow participants to introduce all aspects of the subject. The discussion proceeded in four steps, beginning with an open question: *"What are your associations with the NIP?" *In this step we encouraged the participants to cover parental, professional, organizational and societal aspects. In step two, these aspects were grouped into categories by the participants themselves. The category names (e.g., aim NIP, parents, media) guided answers to the second question: *"How would you describe your role as CVP in relation to the NIP?" *The third step was specifically aimed to reveal participants' beliefs about their role in recommending vaccinations to parents. Therefore, the roles described in step two were used to verify whether participants felt willing and able to perform them, given the fact that recommending vaccinations is one of their tasks. At step four, groups discussed two scenarios regarding changes to the NIP: the recent introduction of universal hepatitis B vaccine (provided only to high-risk groups during the study period) and the possible future introduction of varicella vaccine. We wanted to know whether the participants would still be willing and able to recommend the NIP and what factors were involved in their considerations. The authors pre-tested this four-step design with colleagues.

Each of the four focus groups met once for about 2 hours of discussion during April and May of 2010. The groups were facilitated by a moderator and assistant. The moderator (JMS) was a graduate student at the VU University of Amsterdam with a temporary assignment at the RIVM department of Epidemiology and Surveillance. The assistant (LM) was the RIVM supervisor of the moderator, working as a researcher in the group that evaluates the NIP. In consultation with the other authors, the moderator selected and invited the participants and developed the design and topic list of the focus groups. She also managed the logistics of the meetings.

Data collection for the standard CVPs was considered sufficient with three groups, given time constraints and the emergence of apparent consensus in all groups. One group was considered sufficient to represent the anthroposophical CWCs, whose number is small in the Netherlands.

Each focus group was audio taped, with approval by the participants; flip-charts were used to visualize the topics that had been discussed. The audio-recordings were transcribed by use of digital software of F4 Audiotranskription.

### Analysis

Thematic analysis [21] was performed to identify themes that appeared salient to participants' intention to recommend vaccinations. Analysis was performed separately for the anthroposophical group and the remaining groups, using the qualitative software program MAXQDA (VERBI Software, Germany) version 10.

The moderator and her assistant attended all discussions and examined the resulting transcripts. The moderator processed the data; assigning initial codes to text fragments, refining codes and arranging them in themes and sub-themes. Both researchers validated the themes and sub-themes in relation to the complete dataset. In case of uncertainty, they returned to the original transcripts to discuss the meaning of the citations. The process of coding and development of themes was inductive in nature, as we had not planned beforehand to adhere to a pre-existing theoretical framework [21].

## Results

Virtually all participants were female (one was male), as predominately women work at CWCs. All were physicians, except for three nurses. Their time as CVP ranged from 3 to 41 years and they were diverse in their familiarity with parent populations that varied by educational level, ethnic background and motivation of vaccine refusal or delay (e.g., fear of side effects, religious disapproval, general scepticism regarding vaccination and support of anthroposophy or homeopathy).

Four main themes were extracted from the focus group discussions: 1) Perceived responsibility; 2) Attitudes toward the NIP; 3) Organizational factors; and 4) Participants' relationship with parents. Insights gained are summarized below by theme and sub-theme, with relevant comments by participants. To compare CVPs at standard CWCs and those at anthroposophical CWCs, we treat the former as one group, distinct from the latter. The next few sections refer to the standard CVPs, with the anthroposophical view presented in the final section.

### Perceived responsibility

Participants spoke extensively about what was expected of them, as CVPs, in relation to recommending vaccinations.

#### Formal task perception

They were in agreement that implementation of the NIP was a CVP responsibility and they expected their CVP colleagues to support vaccine uptake:

"The duty of a physician at a child welfare centre is to vaccinate."

"You cannot do this job if you do not support the NIP."

Accordingly, participants provided parents with information about vaccines and possible side effects, in conversation supported by brochures, usually during the first home visit:

"If parents have questions, I believe you have the obligation to explain. I give them the brochure, so they can read it over."

"In most cases, information about vaccination has already been said at the first home visit before they come at the CWC at 4 weeks for the first office visit."

During the visit at 2 months (when vaccination is scheduled to begin), CVPs verified whether parents had any questions left:

"It is my responsibility to check whether parents have enough information before I vaccinate."

### Extra recommendation efforts

The participants recognized that more communication was needed when parents were sceptical about vaccination:

"Some parents doubt or say no because of religious reasons; sometimes you have an extra conversation and I also have a booklet about it, which I give them."

"Nowadays there is a group of parents who are more critical toward vaccination. To them you say: 'Take a brochure, read it carefully and decide afterwards if you want to vaccinate or not. Also take a look at the website of the RIVM, instead of looking only at other critical websites.'"

The participants believed that with parents who were critical toward vaccination, it was their responsibility to ensure that parents made a reasoned decision:

"I always ask their specific reasons for refusing. I believe it is very important that they do not reject it on unfounded grounds."

Some participants recommended making a written inventory of the parents' actual arguments against vaccination:

"If I know their arguments, what their fear is, I will be able to continue from there and explain more about it."

Some participants pursued less discussion with parents who readily accept vaccination:

"Before they visit us for the first time you already know from the home visit whether parents either want to have their child vaccinated or not, and, if I am honest, thereafter little time will be spend talking about the topic if they are accepting."

### Attitudes toward the NIP

Overall, participants were positive about the NIP:

"I tell parents how useful the program is, it is so important; all childhood diseases of a number of years ago have disappeared."

Still, they strongly expressed reservations about projected changes to NIP that might affect their intention to recommend vaccinations:

"If you have doubts yourself about a vaccine, it will become very difficult to be convincing in your information towards parents who are expressing their doubts."

#### Current and future vaccines

Regarding NIP vaccines, participants' main criterion was that the target disease should have a high disease burden. They believed that vaccines in the current program for children of 0-4 years old largely met this criterion. However, concerns were expressed about plans to expand the program to diseases with a relatively low perceived burden:

*"With the current NIP I do not have that feeling*, *i.e*., *doubts as to persuading parents, but I would have that feeling with the vaccine against human papillomavirus and also with varicella. If varicella vaccination would be introduced, I do not know how I should sell this to the parents."*

"Chickenpox is not a serious disease in children. It only gives problems for a few children who belong to a risk group and I wonder whether we should vaccinate the whole population."

Participants were uniformly positive about the extension of hepatitis B vaccine (combined with the DTaP/IPV-Hib vaccine) from high-risk groups to all children, which occurred after the study period:

"We are positive about extending hepatitis B vaccination to all children."

"It makes it for the CVPs more clear and orderly... It becomes easier for us."

*"It is now also a bit unfair*, *i.e*., *its previous limitation to risk groups."*

Participants agreed that vaccine safety was a big point of concern among some parent groups and that side effects were much discussed with parents:

"But the threat of side effects plays an important role for parents who are looking for it."

Side effects I always explain beforehand. Then it does not come so unexpected."

Participants discussed experiences with side effects and safety but agreed that the problem was less worrisome for them than for parents:

"Apparently, side effects are not the first thing we think about."

"Maybe we see side effects as mild compared to parents and their children."

Two injections per consult seemed to be the limit for the participants:

"Otherwise you should vaccinate almost at every consult."

"How many injections can that small body tolerate?"

#### Vaccination schedule

The NIP schedule was valued for being clear, which reduces the chance of errors and makes it easy to explain to parents. Participants differed about program flexibility: whether or how much the schedule might be adapted to individual cases. Some adhered more strictly to the recommended schedule:

"This is the program: you participate or you do not participate. I would not spend too much time on that in talking to parents."

Others provided examples of delayed vaccination:

"Well, you do have parents who believe 2 months is too young to vaccinate; then I say wait for 1 month, think it over."

Some spread out the injections to one per consult, avoiding more than one at a time:

"For example, one consult for DTaP-IPV and another for pneumococcal vaccine and not simultaneously."

Some participants offered parents the opportunity to come back if parents wanted to revise their decision on vaccine refusal:

"Parents are happy when they do not have to decide today. Even if they say no in the first place, if they decide to vaccinate 2 months later, they are still welcome."

### Organizational factors

Organizational factors were defined as the environment of participants, which could facilitate or hinder their recommending vaccinations to parents.

#### Time and other practical considerations

Participants mentioned that CWC consults offered little time to discuss vaccinations with parents:

"Vaccination costs more than the estimated 2.5 min in a consult of 15 min and is not feasible, especially if you need to convince parents about the benefits of vaccination."

Other health checks needed to be completed during the consult as well:

"You just cannot do it all; it is a matter of making choices."

In addition, participants discussed practical constraints that could lead to making mistakes:

"There are many changes, so you might have two types of MMR vaccines in the refrigerator."

"Those hard-to-read packages are irritating anyway!"

"We also regularly have vaccinations with an expired date; this I also do not understand."

#### Information supply

Participants felt they did not always receive timely and objective information from NIP in order to respond adequately to parents' arguments and questions about new or revised vaccination:

"With Hepatitis B this has happened: there was no information about it. I visited a congress and knew I would be alright, but there were a lot of colleagues who thought, 'Next month I have to administer the vaccine and then what should I tell parents?'"

Nor were they told about new regulation and protocols:

"I believe that we are badly kept posted about it. As of first of January 2010, the Haemophilus influenzae type B vaccine is not given anymore after the age of 2 years, i.e., this CVP had just noticed the change."

Or about pertinent scientific research:

*"I expect that we should be kept posted about developments in science" *by receiving *"objective" *information *"on time."*

CVPs also wanted to know more about *"epidemics around us" *and events in the news: *"I believe this is not a minor detail: three children who died just after vaccination against pneumococcal disease. First you hear the whole story from word-of-mouth and media and thereafter you only hear from RIVM that there is no association."*

They also need *"knowledge of what can be found on the internet... what has just been on television."*

*"Somebody from the RIVM should search the internet continuously" *and provide timely and up-to-date information on both sides of a problem:

"That is the balanced information that you want to receive from the RIVM or the government."

#### Interaction with RIVM

Another factor in participants' intention to recommend vaccinations was related to recognition by the RIVM of CVP expertise:

"We cannot exert influence, they just provide a directive to us, you just have to accept it all, but we do have an opinion about vaccines and changes in the program."

One focus group believed CVPs would be more supportive of NIP if the RIVM showed more interest in their arguments and expertise:

"You want to be taken serious as a professional, particularly because you directly experience problems related to the program or practice that directly affect the health care you deliver. If you think this is threatened, you wonder, 'Are we still recognized by RIVM?'"

Some participants felt poorly informed about NIP changes and their context:

"Now, if there are epidemics around us, then the news reaches us very slowly. This can also be due to our executive physician at the CWC, who should forward it to us."

"I believe that we should be informed directly about big changes in the vaccination program."

Several participants who had contacted immunization administration services regarding non-standard situations, like alternative schedules, perceived some inconsistency:

"If you make a call, what you are told to do depends on who is on the phone... Some are being very pragmatic, while others stick to the rules."

### CVP relationship with parents

Establishing a trustful doctor-patient relationship was mentioned as the basis to provide effectively the complete package of care offered at CWCs, including vaccines:

"I believe it is very important that parents have the feeling that the CWC is not only the NIP. If they do not want vaccinations, then maybe you should leave it at that, during the first consult, then continue with it later on. Most important is to establish a trustful relationship so that aside from vaccination a child can make use of the health care provided."

#### Parental types and attitudes

Participants' recommendation practices seemed to be partly determined by perceived types of parents. Several sub-populations of parents were discussed, with most of them accepting the NIP, including migrant parents:

"I noticed that most parents find vaccinating a very logical thing to do."

"Most migrant parents just do it because the doctor says so."

Many parents did not make a deliberate choice but accepted vaccination out of habit:

"Many parents do not think about it, they just do it."

Some mistakenly believe it is mandatory:

"They say, 'Oh, it's not obligated?'"

Some parents have reservations about vaccinations due to contradictory media messages; usually they were concerned about current and future side effects:

"They are afraid of what might happen: 'Will they not say after 20 years that the substance was causing cancer?'"

Other sceptical parents are members or followers of an association critical toward vaccination:

"I believe most critical parents are highly educated, difficult to drive an argument home to, having an own opinion but not always reading the scientific literature."

Others refuse vaccination based mostly on religious grounds or homeopathic grounds:

"But in L. we of course have Reformed Christians, who do not want to vaccinate."

Here we have believers in classical homeopathy, which is a special group for which a CWC provides the vaccine in a sealed phial. They take the sealed phial for the homeopathist or they do not want to vaccinate at all."

"Now, some of them are very convinced, while others can be persuaded."

#### Respect and empathy

Participants believed that they should always respect parents' arguments and their choice to vaccinate their child or not:

"You do not need to have the same opinion to show respect."

"Making parents feel welcome irrespective of their choice of vaccination, while showing that you are positive towards the NIP."

Some participants used terms like *"motivating" *when talking about their way of communicating with parents:

"Motivating is especially one of our tasks."

They stressed the importance of showing empathy towards the arguments of the fearful or sceptical parent:

"I believe that it should start with that: showing empathy towards parents who are afraid of this."

Showing empathy was not always easy, particularly with some arguments or parental attitudes. For example:

"If it is nonsense what the parents are talking about, for example, about autism after MMR vaccination."

"If you indeed feel some hostility, such as 'You do not have to tell me anything.'"

With parents having only moderate concerns about vaccination, participants felt quite competent to establish trust:

"It is just a matter of separating facts and fiction."

"Listening, showing empathy and then discussing your arguments; this will always do."

However, convincing parents was not always possible:

"I have never succeeded to convince somebody who refused vaccination because of religious reasons, despite my efforts."

Communication with members or followers of an association critical towards vaccination was described as difficult and usually ineffective, partly due to parents' closed attitude towards CVPs' information:

"Even facing a close attitude, I always allege in defence of the program."

To some parents who wanted an alternative schedule, an anthroposophical CWC was suggested:

"I also refer to the anthroposophical CWC... Per year, this is 3 times and sometimes 4."

### Anthroposophical view on recommending the NIP

Participants in the anthroposophical focus group were willing to adapt the NIP schedule if requested by the parents, a practice less common at most standard CWCs:

"If they want something else, then we will discuss what is possible. We will not immediately say that this is not allowed."

"Implementing the NIP is the best thing to do from a population view, but they are sitting here as a mother; they have to think what is the best for their child."

Our anthroposophical participants offered parents more elaborate information about vaccines, compared to participants at standard CWCs:

"It is my task to inform them very well; with that information they have to take a decision."

"At the regular CWC, they just say: 'Madam it is time to give the second injection' and the injection needle is already in action, i.e., a humorous exaggeration. Consequently, there is no discussion; then parents visit us with the feeling:'I do not want to vaccinate at all anymore.'"

They described their parent population as diverse, but in general their parents have a more critical attitude towards the NIP than those visiting a standard CWC:

"We are a type of safety net for very critical parents."

"In general they are highly educated, but even if not, they have very well-considered thoughts about their health."

Participants reported parental worries about vaccine safety and, on the other hand, the consequences of not vaccinating their child:

"They have fear for the disease the child could get, fear for side effects, and fear for adapting the schedule."

The participants mentioned that conversations about adapting the program could take a lot of time:

"Most parents do not ask about adaptation, but given those who do our population requires a lot of extra time."

Anthroposophical CVPs saw combined vaccines as restricting the possibilities of flexible implementation of NIP:

*"I believe especially flexibility*, *i.e*., *more than any other factor, would help parents to choose much easier with regard to several vaccinations."*

These participants advocated for continued accessibility to certain monovalent vaccines. Like the other participants, they stressed the importance of a clear scientific basis for any decision to introduce new vaccines. Such a decision should particularly consider the severity of vaccine target diseases. In their view, the pharmaceutical industry and economic considerations sometimes had a strong influence on NIP policy:

"It is not the public servant who decides; it is the pharmaceutical company that develops something whether or not anyone asked for it, and then the public servant says: 'Hey, there is something we might as well use.'"

Another anthroposophical view was that RIVM brochures should not use fear to convince the public to accept vaccination:

"What strikes me is that in general the RIVM language makes people afraid of a disease and then says: 'But we have a vaccine for this; you do not have to be afraid anymore.'"

## Discussion

This is the first study in the Netherlands to provide an overview of CVP attitudes that influence their recommendation of vaccinations to parents.

### Reflection on the main findings

Four main themes were extracted from the focus group discussions. The first could be interpreted as the precondition to CVPs' intention to recommend vaccinations: our sample perceived recommendation as their responsibility. One study [12] described a comparable "professional role" among nurses in the UK who make home visits and bring parents information. Moreover, the Dutch Law on Agreement of Medical Treatment (WGBO) requires CVPs to provide parents with standard information on vaccines. However, a Dutch study [14] showed that some CVPs neglected this responsibility. Our participants at standard CWCs said that many parents accepted vaccinations without need for discussion. Deliberate decision-making was largely stimulated only when parents were reluctant. A study [22] reported that 81% of the Dutch parents made no comparative assessment of vaccination before accepting it. They suggested that because their attitudes are not settled on stable grounds, such parents could be susceptible to misconceptions they find in the social media.

The second theme involved CVP attitudes toward the NIP, which we found largely positive. Similar positivity has been found in various countries [4,11,16]. It is not surprising, as many of our participants reported that you could not do this job if you did not support the NIP. Their main concern was that the target disease should have a high disease burden. In the Netherlands, high disease burden is the first of seven selection criteria used by the Health Council when advising the Minister of Health to introduce a new vaccine [9]. One study [23] found this criterion was important likewise to Minnesota physicians. Many were negative about universal infant vaccination against hepatitis B infection because it has low prevalence in their state. With HPV vaccines, the potential health impact (e.g., adolescent susceptibility to HPV in general, experience diagnosing HPV-related disease in one's practice) made pediatricians hesitant to recommend [24]. Other studies found CVPs concerned about the safety of various vaccines [11,14,23,24], but our participants perceived this as mainly a parental worry. Also, unlike the study described above [24], they did not question vaccine efficacy, perhaps they had confidence in the NIP. They were doubtful only about projected vaccination for diseases with a relatively low burden, which would be difficult to explain and recommend to parents. Accordingly, as reported by one study [25], both CVPs and parents should be well-informed before introduction of a new vaccine: its clinical indications, the official recommendations and the risks and benefits. Interestingly, CVPs at standard CWCs felt that efficient NIP implementation was promoted by a strict schedule and combination vaccines (e.g., combining the hepatitis B vaccine to the DTaP/IPV-Hib vaccine). Similarly, one study [23] found that reluctance regarding universal infant hepatitis B vaccination was due in part to its addition of three more injections to the immunization schedule. However, a strict schedule and combination vaccines were less appealing to anthroposophical CVPs, who wanted to offer the flexibility favoured by a large part of their parent population.

The third theme comprised organizational aspects of NIP that affect recommendation. Previously addressed by the Dutch Health Inspectorate [26] and in several articles, the most discussed were limited time [10,14,27] and incomplete information [14,28]. Time constraints hampered CVP communication with parents during consults. One study [29] reported that among physicians the most commonly reported barrier to discussing vaccination with parents was the time it takes. Participants also mentioned vaccine changes and impractical packaging as time constraints that can lead to mistakes. As for information supply, they wished to be kept up-to-date by the RIVM about media messages on vaccination and about new aspects of the program. From the literature we already know that more CVP knowledge is associated with a higher intention to vaccinate [16,17]. Our participants' request for information suggests a need for CVP workshops or other educational intervention.

The fourth theme involved the relationship between CVP and the parent, with trust being the main basis for communication. On the parents' side, one study [30] found that a trusting relationship with the CVP was central to vaccine acceptance. Our participants described several types of parents with different communication needs. They try to offer respect and empathy but find communication difficult with certain groups and not always effective. One study [24] reported that one of the primary barriers to CVP recommendation of HPV vaccines was the anticipated parental attitudes, e.g., denial that their child would be sexually active and therefore at risk for HPV. Another study [31] ranged parental groups on a continuum scale of "not vaccinating at all" to "accepting vaccines without questions", suggesting diverse communication needs. In one study [29] they reported that physicians in the USA perceived their own spoken words to be the most effective way of convincing sceptical parents. However, the strength of such a message relies on parental trust in the provider's judgment and experience. More knowledge about communication with the spectrum of parents might improve providers' capacities to recommend vaccines.

### Remarks on the study approach

We used the Theory of Planned Behaviour (TPB) mainly as a comprehensive frame of reference for developing the interview scheme. Therefore, our interviews with CVPs accounted for the three constructs of the TPB: *attitude *(toward the NIP and towards perception of the CVP role), *social norms *(expectations of colleagues, staff, RIVM that CVPs should recommend vaccinations) and *perceived behavioural control *(factors perceived to hamper or ease performance of various roles). However, for the analysis we did not reconstruct our data according the TPB constructs. Instead, we chose a more inductive approach of coding, assuming it would allow more open analyses and a richer description of the data. In addition, we wanted to identify and describe sub-themes such that results were closely linked to the original data [21].

As for study limitations, the sampling method might have caused selection bias, attracting a predominance of CVPs with a positive attitude. Second, although others have found that nurse and physician CVPs differ in their intention to recommend the NIP [19,32,33], our findings cannot agree or disagree, because only three nurses participated. Third, the qualitative data might have been influenced by interpretation bias, despite efforts to reduce such bias. These included computer-based analysis, analysis according to a guideline [21] and discussion of the data with the moderators' assistant, not previously involved in the analysis. Fourth, it might be that our data was not fully saturated. As the third focus group reached a point of covering the same topics as the former two groups, we assumed that more groups were unnecessary, especially given our limited study period. Finally, when participants discussed the two scenarios of NIP changes, they did not have a comprehensive overview of related information because not all data were available at the time of the study period. This might have influenced their opinions.

Our design nevertheless resulted in open discussions among participants who were not hesitant to make critical comments. Their response to the study indicated appreciation of the method and gratification to be asked their opinion about vaccine acceptance.

### Future research

We provided an overview of the factors related to participants' intentions to recommend vaccinations. To test the magnitude and relative impact of these factors, questionnaires need to be developed for quantitative analysis among a larger representative group of CVPs. The resulting insights will contribute to a monitoring system that will be installed to oversee the determinants of vaccination acceptance in both CVPs and parents. The system will enable timely intervention when acceptance is seen to be decreasing.

## Conclusions

Our qualitative assessment provides an overview of participants' beliefs associated with the intention to recommend vaccinations to parents. They were motivated to support the NIP, but their willingness and capabilities were affected by the perceived relevance of the vaccines, practical issues of time and knowledge and interaction with diverse types of parents. These results will inform future studies to test the magnitude and relative impact of these factors.

## Competing interests

The authors declare that they have no competing interests.

## Authors' contributions

Under supervision of HdM and LM, JS developed the study design, performed the data collection and data analysis and wrote a draft manuscript. LM assisted during the focus group meetings and contributed to the data analysis and the writing process. LM also finished writing of the manuscript. The study design and focus group design were discussed and constructed with HdM, JvS and TP. All authors contributed to the draft of the final manuscript; their remarks were discussed and processed into the final version that was finally approved by all authors.

## Pre-publication history

The pre-publication history for this paper can be accessed here:

http://www.biomedcentral.com/1471-2458/12/128/prepub
